# Retromolar Triangle Anesthesia Technique: A Feasible Alternative to Classic?

**DOI:** 10.3390/jcm12185829

**Published:** 2023-09-07

**Authors:** Ciprian Ioan Roi, Alexandra Roi, Adrian Nicoară, Alexandru Cătălin Motofelea, Mircea Riviș

**Affiliations:** 1 Department of Anesthesiology and Oral Surgery, “Victor Babeș” University of Medicine and Pharmacy, Eftimie Murgu Sq. No. 2, 300041 Timișoara, Romania; ciprian.roi@umft.ro (C.I.R.); nicoara.adrian@umft.ro (A.N.); rivis.mircea@umft.ro (M.R.); 2 Multidisciplinary Center for Research, Evaluation, Diagnosis and Therapies in Oral Medicine, “Victor Babeș” University of Medicine and Pharmacy, Eftimie Murgu Sq. No. 2, 300041 Timișoara, Romania; 3 Department of Oral Pathology, “Victor Babeș” University of Medicine and Pharmacy, Eftimie Murgu Sq. No. 2, 300041 Timișoara, Romania; 4 Department of Internal Medicine, Faculty of Medicine, “Victor Babeș” University of Medicine and Pharmacy, 300041 Timișoara, Romania; alexandru.motofelea@umft.ro

**Keywords:** dental anesthesia, inferior alveolar nerve block, retromolar triangle anesthesia

## Abstract

Anesthetic techniques play an important role in the outcome of the therapeutic procedures in dentistry. Although inferior alveolar nerve block (IANB) anesthesia is currently the most often used, there are situations that imply the need of an alternative anesthesia technique to overcome the potential risks and complications. The aim of the study was to evaluate the efficacy of the retromolar triangle anesthesia technique in achieving the desired nerve block, while evaluating the duration of the anesthesia for the included cases. Methods: The present prospective study included 50 subjects that had indication of inferior molar extraction. The performed anesthesia technique for these cases was the retromolar triangle approach, and the analyzed parameters for evaluating the efficacy of this anesthesia technique were the positive nerve block of the branches involved in the area (inferior alveolar, buccal, and lingual nerves) and the duration of the anesthesia. Results: The efficiency of the retromolar triangle anesthesia technique was positive in 64% of the cases for the inferior alveolar nerve, 46% of the cases for the lingual nerve, and 22% of the cases for the buccal nerve. The duration of the anesthesia revealed a mean value of 72.4 min, suggesting that the duration is an essential factor in its effectiveness. Conclusions: Retromolar triangle anesthesia can be a viable option for clinicians, offering a simple and easy approach for the management of clinical cases.

## 1. Introduction

Therapeutic procedures in the oral cavity are performed under local anesthesia in the majority of cases. Achieving the proper anesthesia is a desiderate, based on a fundamental knowledge of the nerve anatomy. The most often blocked nerves are represented by the branches of the trigeminal nerve: maxillary and mandibular nerves. In the case of extraction of the inferior molars, the inferior alveolar, lingual, and buccal nerves are considered the “holy trinity” that must be anesthetized during the procedure [1].

The mandibular nerve is the largest branch of the trigeminal nerve and the only one that has motor fibers for the muscles attached to the mandible, the masticatory muscles, the mylohyoid, the anterior belly of the digastric muscle, the tensor veli palatine, and the tensor tympani. It arises from the skull by the oval foramen and divides into two trunks: the anterior one from where the buccal nerve and the motor fibers arise, and the larger posterior trunk from where the auriculotemporal nerve comes out and wraps around the medial meningeal artery, supplying sensory innervation to the cutaneous tissue around the auricula and temporal regions. Furthermore, the posterior trunk divides into two main splits: the lingual and inferior alveolar nerves [1,2].

The inferior alveolar nerve enters in the mandible bone through the mandibular foramen which is located in the center of the inner part of the vertical ramus of the mandible. It has an anterior and descendent path in the mandibular canal until it reaches the mental foramen, where it divides into the incisive and mental nerves. The incisive branch is a terminal branch of the inferior alveolar nerve, which innervates the incisive group, the inferior canines, and the first inferior premolar. The mental nerve arises from the bone and supplies the innervation of the skin over the chin and the mucosa of the lower lip, as well as the buccal gingiva from the midline to the mental foramen [2].

The lingual nerve descends between the lateral pterygoid and the tensor veli palatine. It is situated medial to the inferior alveolar nerve and enters in the floor of the mouth near the lingual face of the inferior wisdom tooth. This nerve supplies the sensitive innervation of the lingual side of the inferior jaw and the mucosa covering the front two-thirds of the tongue [1,2].

For oral surgery interventions in the mandibular area, block anesthesia is used instead of infiltration anesthesia due to the high bone mineral density, limited access to the inferior alveolar nerve, and high incidence of anatomical variations. The classic inferior alveolar nerve block (IANB) on the lingula is called the Spix technique [3]. This is the most common technique used for the simultaneous anesthesia of both the inferior alveolar and the lingual nerves. The anesthetic substance is deposited above the mandibular foramen before the entrance of the nerve in the mandibular canal. This intraoral approach has a success rate from 71% to 81% [4]. Conventional anesthesia is based on the dental needle insertion near the mandibular foramen, where the inferior alveolar nerve is located before it enters in the mandibular canal. The anesthesia landmarks used for this technique are represented by osseous landmarks (temporal crest of the vertical ramus of the mandible), dental landmarks (inferior molars occlusal plane), and soft-tissue landmarks (pterygomandibular raphe). The needle insertion site is located 1 cm superior from the inferior molar occlusal plane, posterior from the temporal crest, and anterior from the pterygomandibular raphe. Needle insertion is continued until bony resistance is felt. Touching the bone with the needle must be very gentle, as this causes pain by stinging the periosteum [5].

Being a deep technique, several accidents can occur during anesthetic administration: hemorrhage with hematoma formation, intravascular injection, trismus, nerve damage, needle breakage, and pain [6,7,8].

Research reveals that the most important factor explaining the failure of the IANB is the operator’s defective technique [9,10]. However, other secondary anatomic factors can explain the failure: anatomic variations such as accessory nerve supply by mylohyoid nerve, cervical cutaneous nerve C1/C2, auriculotemporal nerve, variable course of nerve, collateral anastomosis of the nerves, variation in mandibular foramen position, bifid alveolar nerve or bifid mandibular canal, and presence of the retromolar foramen; different pathologies near the mandibular foramen or at the needle insertion site: trismus, infection, inflammation, previous surgeries; psychological factors such as fear, anxiety, and apprehension [11,12,13,14,15,16].

Many alternative approaches that modify the IANB technique have been described in the scientific literature, all of which are aimed at achieving a high success rate, and reducing the risk of hemorrhage, intravascular injections, and nerve damaging [17,18,19,20,21,22,23]. The conventional alternatives when the anesthesia of the inferior alveolar nerve is not installed are reported to be second administration of the IANB, buccal infiltration near the apex of the molars, intraligamentary anesthesia technique, intraosseous anesthesia, intrapulpal injection, simultaneous techniques such as the Gow–Gates mandibular nerve block, closed-mouth block (Vazirani–Akinosi block), or other techniques that modify the classic anesthesia.

The retromolar triangle is a triangular area located distal of the inferior last molar. This space is formed by the fork in the temporal crest, located in the internal face of the mandibular ramus and distal face of the last mandibular molar [3]. The bone in this area has an anatomic particularity, being perforated by a variable number of foramens with various sizes in diameter that allow the buccal artery to make anastomoses with the inferior alveolar vessels in the mandibular canal. On the basis of this particularity, the local anesthetic solution deposited in this area can diffuse and reach the inferior alveolar nerve through this communication between the retromolar triangle and the mandibular canal [24].

Our study is focused on the presentation and evaluation of the retromolar triangle anesthesia technique. This alternative technique that blocks the inferior alveolar nerve, while inconstantly blocking the lingual and buccal nerves, was described by Suazo Galdames as an alternative option for cases involving patients with blood disorders.

## 2. Materials and Methods

The purpose of the present prospective study was to test the efficiency of the anesthesia technique in the retromolar triangle for the block of the inferior alveolar nerve and its terminal branches for the extraction of the inferior molars.

Our study was approved by the Ethics Committee of “Victor Babeș” University of Medicine and Pharmacy Timișoara (no.18/2023), and patients agreed and signed an informed consent form that followed the guidelines of the Declaration of Helsinki.

### 2.1. Recruitment

In this study, we included patients on the basis of the following inclusion and exclusion criteria:

Inclusion criteria:

- Age: 18–50 years.
- Both males and females.
- Inferior molars with indication of exodontia: teeth with decays unrestorable by odontal therapy, apical pathology, severe periodontal diseases, orthodontic extractions, pre-prosthetic extractions.

Exclusion criteria:

- Age: <18 years.
- Pregnancy.
- Patients with acute pulpitis of the inferior molars.
- Patients with odontogenic abscesses.
- Patients with tumors of the intermaxillary commissure.
- Patients with contraindications to the administration of anesthetics with adrenaline or articaine.
- Patients with mucosa lesions on the injection site.

After applying the inclusion and the exclusion criteria, 50 patients were included in the study: 35 males and 15 females in the age range of 18–49 years.

### 2.2. Armamentarium

- Short needle: 21 mm, 30 gauge diameter (Septoject, Septodont, Saint-Maur-des-Fosses Cedex, France).
- One ampoule: 1.7 mL of articaine with 1:100,000 adrenaline (Septanest, Septodont, Allington Kent, UK).
- Chlorhexidine solution (Eludril, Pierre Fabre, Lavaur, France).
- Electric pulp test (DenjoyDental Co., Ltd., Changsha, China),
- Periodontal probe.

### 2.3. Patient Position

- The pulp test checks the vitality of the first/second lower molar on the vestibular face and notes the intensity to which it responds.
- The inferior occlusal plane is parallel with the Camper plane.

### 2.4. Doctor Position

- For the fourth quadrant: 8–9 o’clock position.
- For the third quadrant: 9–10 o’clock position.
- The left hand removes the soft tissues.
- Direct visibility of the puncture site.

### 2.5. Vitality Tests

The oral cavity is wide open and the pulp sensibility of the tooth that has indication of extraction is measured according to the producer indications.

### 2.6. Insertion Needle Area (Figure 1)

- The oral mucosa of the area of the retromolar triangle.
- At 5 mm distal from the distal face of the last molar.
- In the most central part of the lateral retromolar triangle.

**Figure 1:**
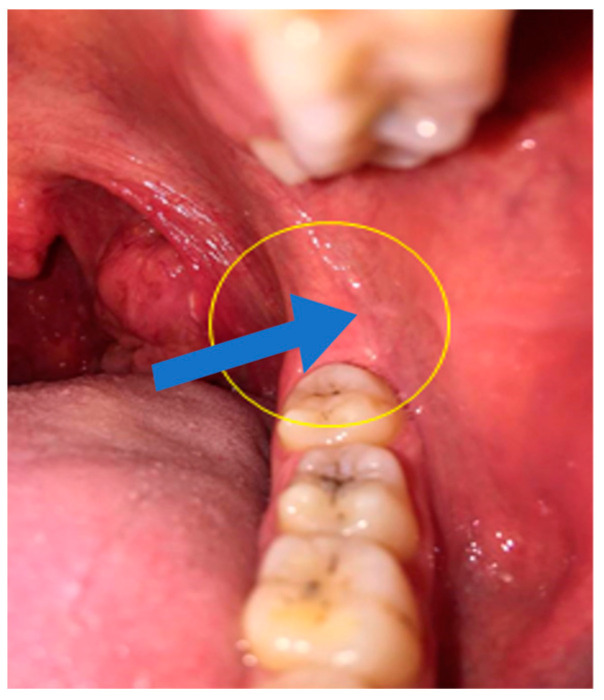
Insertion needle area.

### 2.7. Needle Level

- Perpendicular to the mandibular bone.
- Rearward, outward, and downward orientation.

### 2.8. Anesthesia Technique (Figure 2)

- The puncture site must be dried with a sterile cotton gauze.
- A chlorhexidine-based antiseptic solution is applied.
- Local anesthesia with topical anesthetic (benzocaine 20%) is performed (Opahl, USA).
- The syringe is drawn parallel to the occlusal mandibular plane.
- The needle is inserted in the mucosa, searching for bone contact, after which the anesthetic solution is slowly deposited (1 mL/min).

**Figure 2:**
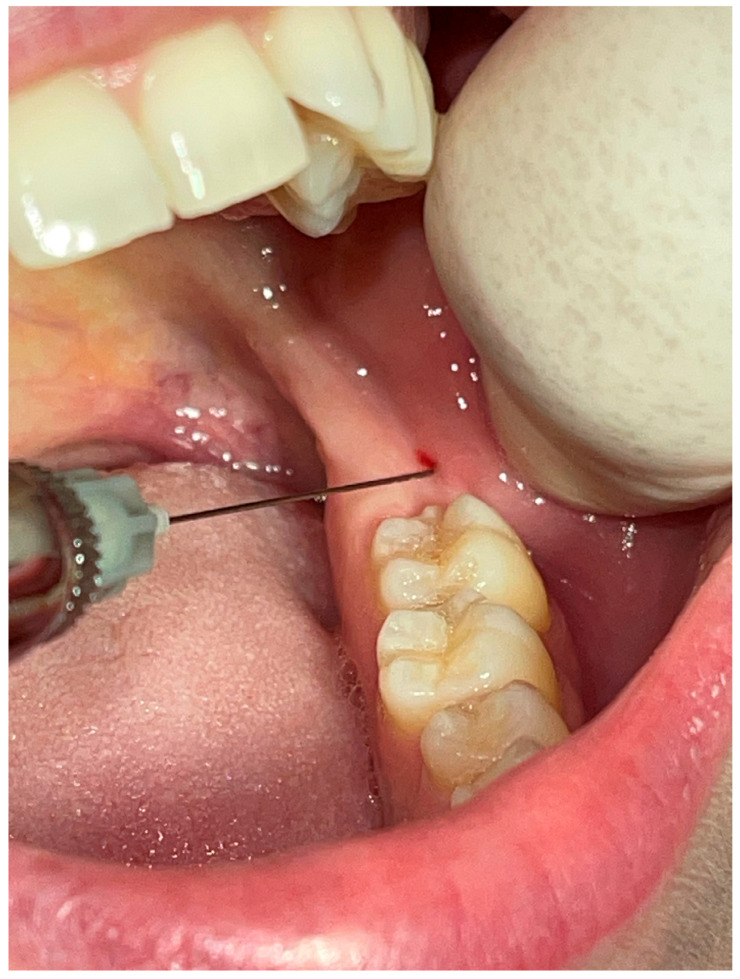
Anesthesia technique.

### 2.9. Anesthesia Instalation and Check

- For inferior alveolar nerve: With the pulp test, we checked after 15 min the anesthesia on the same first/second inferior molars that were initially tested by measuring the intensity of the stimuli necessary for the tooth pulp to respond. The guide values followed by the manufacturer were 0–40 for the normal reaction of the dental pulp, 40–80 for partial anesthesia, and over 80 for installed anesthesia of the tooth pulp. The value was noted.
- For mental nerve: With the periodontal probe, the mucosa of the lower lip was punctured in front the inferior canine on the anesthetized side.
- For buccal nerve: With the periodontal probe, the vestibular mucosa was punctured in front of the first molar on the lower anesthetized side.
- For lingual nerve: With the periodontal probe, the lingual mucosa was punctured on the lingual gingival sulcus of the first molar on the lower anesthetized side.
- The results for mental, buccal, and lingual nerves were noted using the visual analog scale for pain (VAS).

### 2.10. Statistical Analysis

Continuous data are presented as the mean with SD if the data are normally distributed, and as the median with the 25th and 75th percentiles for nonparametric data. Categorical data are summarized as counts and percentages. Linear regression analysis was used to examine the relationship between post-anesthesia values (dependent variable) and other variables including VAS pain scores (independent variables). The regression coefficient (R), R^2^ (coefficient of determination), and *p*-values for individual predictors were calculated assess the strength of association between variables. A *p*-value less than 0.05 was considered statistically significant. Differences between groups for continuously normal data were tested using Welch’s *t*-test for two groups or ANOVA if there were more than two groups. Nonparametric continuous data were tested using a Mann–Whitney U test for two groups or a Kruskal–Wallis test for three or more groups. Differences between categorical data were tested using the test or Fisher’s exact test when the expected cell count was less than five. Continuous variable distributions were tested for normality using the Shapiro–Wilk test. Sample-size calculation was performed prior to the study, aiming to provide a confidence level of 95% and a statistical power of at least 80%. All statistical analysis was performed with R (version 3.6.3) using the Tidyverse, Finalfit, MCGV, Stringdist, Janitor, and Hmisc packages.

## 3. Results

### 3.1. Patients’ Sex and Age Distribution

The data represented a sample of 50 individuals, with a male predominance (70%). The age of the participants ranged from 18 to 41 years, with an average age of 27.5 years, as presented in Table 1.

### 3.2. Extracted Teeth

The teeth involved in the present study are presented in Table 2. The inferior first molars were the most involved teeth with exodontia indication.

### 3.3. Retromolar Triangle Anesthesia Efficiency

This alternative anesthesia, performed in the retromolar triangle, was efficient in 32 cases (64%) out of 50 patients for the inferior alveolar nerve IAN, 11 cases (22%) out of 50 patients for the buccal nerve, and 23 cases (46%) out of 50 patients for the lingual nerve (Table 3).

The duration of anesthesia had a mean of 72.4 min, but it is important to note that this value was significantly different between cases where anesthesia worked (mean of 113.1) and where it did not (mean of 0.0), with a *p*-value less than 0.001. This indicates that the duration of anesthesia is a significant factor in its effectiveness. The VAS (visual analog scale) scores for lingual, buccal, and mental nerves showed some variation, but the most notable difference was seen in the VAS mental score between cases where anesthesia worked (mean of 0.6) and where it did not (mean of 7.1), with a *p*-value less than 0.001. This suggests that the VAS mental score is a significant predictor of anesthesia effectiveness. The post-anesthesia value also significantly differed between cases where anesthesia worked (mean of 78.2) and where it did not (mean of 52.1), with a *p*-value less than 0.001. This further supports the effectiveness of the anesthesia.

The data also provide information on the specific teeth extracted, but there was seemingly no significant association between a specific tooth and anesthesia effectiveness. All data are presented in Table 4.

**Figure 3:**
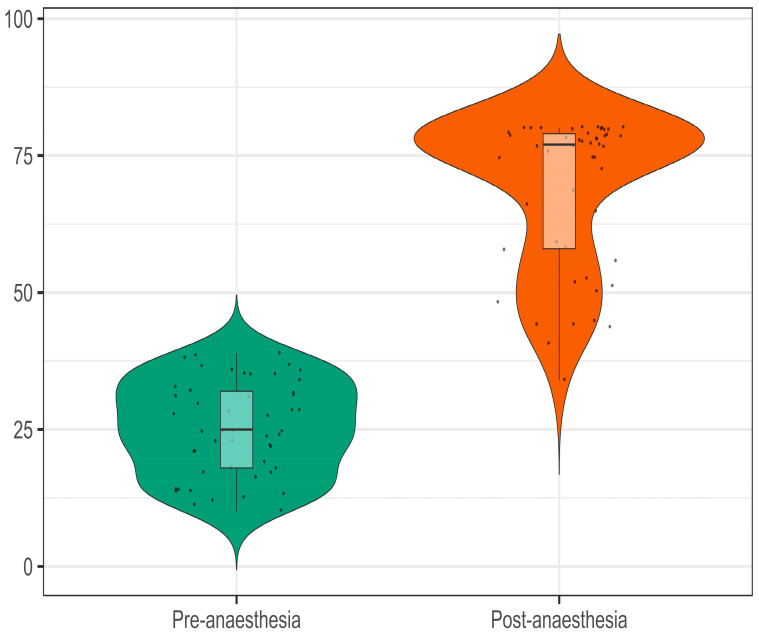
Pulp test measures before and after the anesthesia.

The initial value, presumably a measure before anesthesia with the pulp test, had a mean of 25.3, while the post-anesthesia value significantly increased to a mean of 68.8, as shown in Figure 3. This suggests that the anesthesia had a substantial effect on this measure. The guide values followed by the manufacturer of the pulp test were 0–40 for the normal reaction of the dental pulp, 40–80 for partial anesthesia, and over 80 for installed anesthesia of the tooth pulp.

In our model, R was estimated to be 0.9452, indicating a strong positive linear relationship between the predictor variables and the post-anesthesia values, and R^2^ was estimated to be 0.8933, indicating that approximately 89.33% of the variance in the post-anesthesia values could be explained by the predictor variables.

The coefficient for VAS mental was estimated to be −3.91945. It was statistically significant (t = −19.5316, *p* < 0.001), suggesting that, for every unit increase in VAS mental, the post-anesthesia values decreased by approximately 3.92 units (Table 5). This indicated a strong negative association between VAS mental and the post-anesthesia values.

## 4. Discussion

Wide categories of oral treatments in the mandibular region require the anesthesia of the inferior alveolar nerve, starting with decay therapies, followed by endodontic and periodontal treatments, and ending with oral surgery procedures such as tooth extractions, odontectomies, apicectomies, or surgical removal of the odontogenic cysts. Adequate anesthesia for pain control is often difficult to achieve in this specific area. Local infiltration techniques are performed with high success rates in the upper maxilla but seem to have disappointing results when performed in the lower molar region. A few factors that can explain this theory are the thickness of the cortical bone which is reinforced by the external oblique ridge (a crest extending from the ramus to the body of mandible that provides the insertion point for the muscles), the mineral bone density, and the various number of the structures that must be penetrated by the local anesthetics to achieve the inferior alveolar nerve branches (central core theory) [25].

The clinical use of the classic IANB technique addressed to the mandibular lingula is, in the majority of cases, the first choice during invasive dental procedures that are performed in the area of the posterior mandibular teeth, especially molars and second premolars. The local anesthetic must be placed as rear as possible near the inferior alveolar trunk, before the nerve entering through the mandibular foramen in the mandibular canal. Even with a standardized anesthetic technique, not all IANBs performed are successful. IANB has a failure rate between 29% and 39%, and clinical studies have reported even higher failure rates of 44% to 81% in mandibular molars with irreversible pulpitis [16,26,27,28].

The higher failure rate of the IANB technique can be explained by a number of factors. First of all, the location of the mandibular foramen, which is the most important landmark where the local anesthetic substance must be deposited, can be influenced by the mandibular skeletal characteristics, the deviation of condylar length, the occlusal plane, or the age of the patients [29]. Only after a rigorous localization of the mandibular foramen can the needle insertion site be determined. Secondly, the anatomical variations of the inferior alveolar nerve can influence the installation of the anesthesia. We remind here the presence of the accessory mylohyoid nerve, bifid mandibular nerve, retromolar foramen, or molar teeth accessory innervation by the buccal nerve and great auricular nerve. In these situations, pain management cannot be resolved by repeating the traditional anesthesia technique at lingula. Clinically, it can be diagnosed when numbness of the lower lip is present but the tooth is still responding sensitively when stimulated [12].

A series of alternative local anesthesia techniques for the inferior alveolar nerve were designed in order to decrease the high failure rate or risks and complications. It is well known that the traditional technique is contraindicated in children or in patients with blood dyscrasias, or in chronic anticoagulant treatment due to the intravascular puncture with hematoma formation [30].

The mandibular bone in the retromolar triangle is perforated by a variable number of foramens with different sizes, which allow the anastomosis of the buccal artery with the inferior alveolar vessels in the mandibular canal. Deposition of local anesthetic solution in this area can reach the inferior alveolar nerve through this communication between the retromolar triangle and the mandibular canal [3,24]. The anesthesia of the inferior alveolar nerve in the retromolar triangle was introduced and applied in dentistry, being preferred due to the advantages offered: the visibility of the needle insertion site, which can be observed during the anesthetic deposition; the higher success rate of the anesthesia technique; the very low risk of blood vessel puncture because no major arteries or veins are located in the area; a reduction to minimum in the intravascular deposit of anesthetic substances; near-zero hematoma formation.

In our present study, we obtained an anesthesia efficiency of the inferior alveolar nerve in 64% of the cases, a few percent lower than the data available in the scientific literature [3,31]. These data suggest the efficacity of this technique and the fact that it can be used in dental practice in a wide range of therapeutic treatments of the inferior molars and premolars. Moreover, taking into consideration that retromolar triangle anesthesia is not a deep technique, it can be successfully used in patients with different systemic diseases that have contraindications for deep anesthetic techniques, or for whom the potential complications and accidents are life-threatening.

With this technique, the anesthesia of the buccal mucosa from the inferior third molar to the first premolar was obtained in 22% of the cases. The anesthesia of the buccal nerve could be explained by the anatomical position and proximity of the retromolar triangle. Furthermore, the anesthesia of the lingual nerve was obtained in 46% of the cases, due to the same reasons.

The results of the anesthesia efficiency were validated taking into consideration both objective and subjective values. For the objective installation of the anesthesia in the retromolar triangle, we measured the pulp sensibility and reaction before and after performing the anesthesia, with the help of an electric pulp test. According to the existing scientific data, the electric pulp test is a valuable tool for the clinical evaluation of the pulp response and can be used as an indicator of local anesthesia installation or in predicting potential anesthetic problems [32]. Additionally, prolonged electric pulp testing causes no histological damage to the dental pulp, being a safe procedure. To be more accurate, we evaluated the installation of the anesthesia of the inferior alveolar nerve, by exploring with the periodontal probe the mucosa of the lower lip and puncturing in front of the inferior canine on the anesthetized side. It is well known that the mental nerve is a terminal branch of the inferior alveolar nerve. The subjective evaluation of the anesthesia installation was performed using the visual analog scale (VAS). Numerous methodologies and tests exist to assess the pain that originates from the oral cavity. It is well recognized that it is extremely difficult to quantify pain due to its subjective nature. The VAS has the advantage of simple usage and an unlimited number of possible responses [33]. The VAS mental score in our study was a significant predictor of anesthesia effectiveness in the retromolar triangle.

Regarding the duration of the anesthesia, our study reported a mean of 72.4 min. This duration of anesthesia is sufficient for the majority of therapeutic treatments in the posterior mandibular region and is similar to the data reported in the scientific literature for classic troncular techniques [34]. This idea supports that, during anesthesia in the retromolar triangle, the anesthetic solution disseminates from the injection site to the mandibular canal, where the inferior alveolar nerve is located.

## 5. Conclusions

Effective anesthesia in the dentistry field allows the clinician to perform accurate treatments under a proper control. Taking into consideration that there are situations when deep block anesthesia has contraindications, an alternative technique is welcome. The retromolar triangle anesthesia technique proved to be a desirable approach, achieving a satisfactory anesthetic result that overcomes the potential risks and complications of block anesthesia. Offering the perspective of an accessible injection site, a wide territory to be anesthetized, and a sufficient duration of the anesthesia, the retromolar triangle anesthetic technique can be a reliable option for the future.

## Figures and Tables

**Table 1:** Patients’ sex and age distribution.

Overall (N = 50)	Data
Male	35 (70.0%)
Female	15 (30.0%)
Age range	18–41 years
Age mean (SD)	27.5 (6.5)

**Table 2:** Teeth with extraction indication distribution.

Overall (N = 50)	Data
Left first mandibular molar: 3.6	12 (24.0%)
Left second mandibular molar: 3.7	4 (8.0%)
Left third mandibular molar: 3.8	9 (18.0%)
Right first mandibular molar: 4.6	10 (20.0%)
Right second mandibular molar: 4.7	8 (16.0%)
Right third mandibular molar: 4.8	7 (14.0%)

**Table 3:** Anesthesia efficiency.

Anesthesia Efficiency	Overall (N = 50)
Anesthesia worked (IAN)	32 (64.0%)
Anesthesia did not work (IAN)	18 (36.0%)
Anesthesia worked (BUCCAL N.)	11 (22.0%)
Anesthesia did not work (BUCCAL N.)	39 (78.0%)
Anesthesia worked (LINGUAL N.)	23 (46.0%)
Anesthesia did not work (LINGUAL N.)	27 (54.0%)

**Table 4:** Anesthesia efficiency aspects.

Dependent: Anesthesia Efficiency		Anesthesia Did Not Work	Anesthesia Worked	Total	*p*
Anesthesia duration	Mean (SD)	-	113.1 (18.0)	72.4 (56.7)	<0.001
VAS mental	Mean (SD)	7.1 (1.6)	0.6 (0.8)	2.9 (3.4)	<0.001
Post-anesthesia value	Mean (SD)	52.1 (9.4)	78.2 (1.9)	68.8 (13.9)	<0.001
Pre-anesthesia value	Mean (SD)	22.9 (9.4)	26.6 (7.9)	25.3 (8.6)	0.153
Tooth	3.6	3 (16.7)	9 (28.1)	12 (24.0)	0.511
	3.7	3 (16.7)	1 (3.1)	4 (8.0)	
	3.8	4 (22.2)	5 (15.6)	9 (18.0)	
	4.6	3 (16.7)	7 (21.9)	10 (20.0)	
	4.7	2 (11.1)	6 (18.8)	8 (16.0)	
	4.8	3 (16.7)	4 (12.5)	7 (14.0)	
VAS buccal	Mean (SD)	4.8 (2.5)	5.2 (2.7)	5.1 (2.6)	0.624
VAS lingual	Mean (SD)	3.6 (2.5)	3.3 (3.0)	3.4 (2.8)	0.721
Age	Mean (SD)	27.6 (6.8)	27.5 (6.4)	27.5 (6.5)	0.964
Sex	M	14 (77.8)	21 (65.6)	35 (70.0)	0.563
	F	4 (22.2)	11 (34.4)	15 (30.0)	

**Table 5:** Model coefficients—post-anesthesia values.

Predictor	Estimate	SE	t	*p*
Intercept	80.11821	1.8248	43.9051	<0.001
VAS mental	−3.91945	0.2007	−19.5316	<0.001
VAS buccal	−0.03863	0.2560	−0.1509	0.881
VAS lingual	0.11731	0.2400	0.4888	0.627

## Data Availability

Data supporting the reported results can be provided on request.
